# ARTEMIS: A Novel Mass-Spec Platform for HLA-Restricted Self and Disease-Associated Peptide Discovery

**DOI:** 10.3389/fimmu.2021.658372

**Published:** 2021-04-23

**Authors:** Kathryn A. K. Finton, Mi-Youn Brusniak, Lisa A. Jones, Chenwei Lin, Andrew J. Fioré-Gartland, Chance Brock, Philip R. Gafken, Roland K. Strong

**Affiliations:** ^1^ Division of Basic Science, Fred Hutchinson Cancer Research Center, Seattle, WA, United States; ^2^ Clinical Research Division, Fred Hutchinson Cancer Research Center, Seattle, WA, United States; ^3^ Proteomics Shared Resource, Fred Hutchinson Cancer Research Center, Seattle, WA, United States; ^4^ Vaccine and Infectious Disease Division, Fred Hutchinson Cancer Research Center, Seattle, WA, United States

**Keywords:** MHC class I, peptide-HLA complex, mass spectrometry, immunotherapy, ligandome analysis

## Abstract

Conventional immunoprecipitation/mass spectroscopy identification of HLA-restricted peptides remains the purview of specializing laboratories, due to the complexity of the methodology, and requires computational post-analysis to assign peptides to individual alleles when using pan-HLA antibodies. We have addressed these limitations with ARTEMIS: a simple, robust, and flexible platform for peptide discovery across ligandomes, optionally including specific proteins-of-interest, that combines novel, secreted HLA-I discovery reagents spanning multiple alleles, optimized lentiviral transduction, and streamlined affinity-tag purification to improve upon conventional methods. This platform fills a middle ground between existing techniques: sensitive and adaptable, but easy and affordable enough to be widely employed by general laboratories. We used ARTEMIS to catalog allele-specific ligandomes from HEK293 cells for seven classical HLA alleles and compared results across replicates, against computational predictions, and against high-quality conventional datasets. We also applied ARTEMIS to identify potentially useful, novel HLA-restricted peptide targets from oncovirus oncoproteins and tumor-associated antigens.

## Introduction

The mammalian immune system surveils cellular proteomes through recognition of peptide fragments of endogenous proteins bound to extracellular HLA class one proteins (HLA-I), thus detecting intracellular infection or transformation events (1). These HLA-I bound peptides, mostly eight to 14 residues long, are presented on the cell surface for recognition by, for example, αβ TCRs on cytotoxic CD8+ T cells (2). There are up to ~10^4^ distinct peptides presented on the surface of a typical cell distributed across up to ~10^5^ HLA-I/peptide complexes (pHLAs) (3, 4), constituting the HLA-I-restricted “*ligandome*” (5). Therefore, a cellular ligandome only represents a tiny percentage of all possible proteome-derived peptides. Peptides from self-proteins populate the ligandome in the absence of disease but peptides from pathogen or tumor proteins are added during infection or cancer. T cell responses to self-peptide pHLAs can also be involved in the initiation and progression of autoimmune diseases (6). The ligandome is highly unevenly distributed across constituent peptides, temporally dynamic, and affected by cell type, the cellular environment, disease state, and the peptide specificity of the HLA alleles comprising the cellular haplotype. The full repertoire of HLA-I presentable peptides across ligandomes is termed the “*presentome*” (1). From a basic science perspective, cataloging ligandome/presentome repertoires is fundamental for understanding antigen processing, editing, and presentation (7); how self is defined; how immune tolerance to self is broken; how the immune system recognizes and responds to disease; and tumor/pathogen immunoevasion mechanisms. From a translational perspective, the ability to identify and define disease-specific HLA-I restricted peptides enables diagnosis and treatment. Many potent cancer immunotherapies target HLA-I presented peptides derived from oncogenic viruses, tumor-specific mutations (neoantigens), or aberrantly expressed tumor-associated proteins, yielding exquisitely focused treatments (8).

Methods to identify HLA-I restricted peptides across ligandomes or from specific proteins fall into three broad categories: heuristic computational prediction [e.g. NetMHCpan (9, 10)]; testing candidate peptides in *in vitro* T cell activation assays (e.g. ELISPOT); and immunoprecipitation (IP) of pHLAs from detergent-solubilized membrane fractions, acid-eluting peptides, and sequencing by mass spectroscopy (MS) (11). However, computational prediction methods can have high false-positive and false-negative rates (12, 13), which we further confirmed. ELISPOT and related techniques, which rely on TCRs as specificity reagents, can be confounded by inherent TCR polyspecificity, the potential disconnect between TCR/pHLA *binding* and T cell *activation*, and the potential for cellular processing of the input peptide (“trimming”) *in vitro* during presentation (14–17). MS techniques require adequate quantities of the biological sample, sophisticated instruments and workflows, and complex analysis and deconvolution of results (18), and can be confounded by contaminants, particularly detergents. MS IP results using pan-HLA antibodies, a standard approach (11), requires computational binning and allele-assignment of peptides, assuming that an observed peptide binds only one allele in a haplotype, which may not be valid across HLA supertypes (19, 20). [A recent workaround for this problem involves the painstaking introduction of single HLA alleles one-by-one stably into an HLA-negative cell line for MS IP analyses (21).] Additionally, we showed that false-positive rates in computational predications were elevated by overprediction of allele assignments.

We have addressed many of these limitations with ARTEMIS: a simple, robust, and flexible platform for peptide discovery across ligandomes, optionally including specific proteins-of-interest, that combines novel, secreted HLA-I discovery reagents spanning multiple alleles, optimized lentiviral transduction (22), and streamlined affinity-tag purification protocols to improve upon conventional MS IP methodology. This platform fills a middle ground between existing techniques: sensitive and adaptable, but easy and affordable enough to be widely employed and to incorporate high-order replicate analyses. In order to fully validate this platform, we used ARTEMIS to catalog allele-specific ligandomes for seven HLA alleles (HLA-A*02:01, HLA-A*03:01, HLA-A*11:01, HLA-A*24:02, HLA-B*07:02, HLA-B*15:01, and HLA-C*07:02) from human HEK293 cells and compared results across replicates, against NetMHCpan predictions, and against high-quality conventional (IP) (11) and single-allele (sIP) (21) results. Initial HLA alleles were selected for study based on maximum comparative value with previous results and global population coverage. We also applied this methodology to identify potentially useful HLA-restricted peptide targets from oncovirus oncoproteins including Human Papilloma Virus (HPV) 16 E6/E7 (23) and Merkel Cell Polyomavirus (MCV) large T antigen (24), from the tumor associated-antigen mesothelin (MSLN) (25), and from an HIV Env gp140.

## Materials and Methods

### SCD Expression, Purification, and Peptide Isolation

HLA sequences were engineered into SCDs by replacing the native β_2_m leader peptide with a murine Igk leader (METDTLLLWVLLLWVPGSTG) and fusing the β_2_m sequence to a (G_4_S)_4_ linker, the native HLA heavy chain ectodomain sequence, a GGS linker, and a hexa-histidine purification tag. cDNAs encoding SCDs and target proteins were codon optimized for human cells (Genscript), synthesized (Genscript), and subcloned into optimized lentiviral vectors (22) incorporating either mCherry (SCD) or GFP (target protein) fluorescent reporter proteins (26). SCD and target protein co-transductions were carried out as previously described (22) with near 100% efficiencies as judged by reporter fluorescence. Target proteins included the E6 and E7 oncoproteins of the HPV16 high-risk strain (GenBank AAD33252.1, AAO85409.1), the truncated form of MCV LT associated with cancer (27), the human MSLN precursor fusion protein (MPF+MSLN, GenBank AAV87530.1), and the HIV Env gp140 construct from strain SF162 (28). HEK293 Freestyle cells (Invitrogen, catalogue number R79007, RRID : CVCL_D6642) were grown in Freestyle 293 Expression media (Gibco, catalogue number 12338018) with shaking at 130 rpm, 37° C, 8% CO_2_ in vented shake flasks. Cells were transduced at a density of 10^6^ cells/mL in 10 mL Freestyle media, incubated overnight, and 20 mL fresh media was added the following day. 2.0 ng/mL IFNγ (Thermo Fisher, catalogue number RP-8607) and 3.0 ng/mL TNFα (Cell Applications, catalogue number RP1111-100) were added when the culture reached 0.5 x 10^6^ cells/mL in 100 mL. Cultures were harvested once densities reached ~8 x 10^6^ cells/mL in 200 mL total culture volume. SCD yield was assessed by Western blot using a XCell II blot module (Thermo Fisher), THE HIS mAb (GenScript, catalogue number A00612), and LumiGLO peroxidase chemiluminescent substrate kit (Seracare, catalogue number 5430-0040). Cells were pelleted and the supernatant was filtered, supplemented with 150 mM NaCl, incubated with 200 μL Ni-NTA agarose (Qiagen, catalogue number 30210), applied to a gravity flow column, and washed with 10 column volumes of PBS. Peptides were eluted from column-bound pHLAs with 5 M guanidinium HCl, 250 mM NaCl, 50 mM NaPO_4_, 1 mM DTT (pH = 8). [Addition of reducing agents is crucial for efficient recovery of cysteine-containing peptides.] Eluted peptide purity (i.e., absence of SCD) was assessed by Western blot. Samples were desalted using an Oasis HLB cartridge (Waters), eluted with 30% v/v acetonitrile, 0.1% v/v TFA, and lyophilized.

### Mass Spectrometry

Peptides were analyzed on either hybrid Orbitrap Elite ETD or tribrid Orbitrap Fusion mass spectrometers (Thermo Fisher). On Elite instrumentation, desalted peptides were resuspended in 2% v/v acetonitrile, 0.1% v/v formic acid, and 1 mm dithiothreitol, and analyzed by liquid chromatography-electrospray ionization MS with an Easy-nLC II nano-flow liquid chromatography system (Thermo Scientific) coupled to the Elite mass spectrometer using a trap-and-column configuration. Peptides were desalted inline on an RPC trap column (100 mm × 20 mm) packed with Magic C_18_AQ (Michrom Bioresources 5 mm 200 Å resin) and separated with an RPC column (75 mm × 250 mm) packed with Magic C_18_AQ (Michrom Bioresources 5 mm 100 Å resin) directly mounted on the electrospray ionization source. Peptide elution was carried out using a 90-minute gradient from 7% to 35% v/v acetonitrile plus 0.1% v/v formic acid at a flow rate of 400 nL/minute. Capillary temperature was set to 300° C and a spray voltage of 2750 Volts was applied. The Elite mass spectrometer was operated in the data-dependent mode, switching automatically between MS survey scans in the Orbitrap (AGC target value 1,000,000, resolution 240,000, and injection time of 250 milliseconds) with MS/MS spectra acquisition in the linear ion trap (AGC target value of 10,000 and injection time of 100 milliseconds). The 20 most intense ions from the Fourier-transform full scan were selected for fragmentation in the linear trap by collision-induced dissociation with a normalized collision energy of 35%. Selected ions were dynamically excluded for 15 seconds with a list size of 500 and an exclusion mass width of ±0.5 Daltons. Elite data were analyzed using Proteome Discoverer 1.4 (Thermo Scientific) searching against a 2014 Uniprot human database that included common contaminants (29). A no-enzyme search was performed with a minimum peptide length of six residues and a maximum length of 144 residues. The precursor ion tolerance was set to 10 ppm and the fragment ion tolerance was set to 0.8 Daltons. Variable modifications included oxidation on methionine (+15.995 Daltons) and carbamidomethyl on cysteine (+57.021 Daltons).

On Fusion instrumentation, peptides were either brought up in 2% v/v acetonitrile, 0.1% v/v formic acid and analyzed as-is or fractionated using a high-pH RPC spin cartridge, with fractions collected at 5%, 7.5%, 10%, 12.5%, 17.5%, 20%, and 50% v/v acetonitrile, 0.1% v/v triethylamine. Fractions were taken to dryness and resuspended in a solution containing 2% acetonitrile (v/v), 0.1% formic acid, and 1 mM dithiothreitol just prior to MS analysis. MS analyses were performed with a Thermo Scientific Easy-nLC 1000 nano-flow liquid chromatography system (Thermo Scientific) coupled to the Fusion mass spectrometer using a trap-and-column configuration and a column heater set at 40° C. Chromatographic separations were carried out using a 90-minute gradient from 0% to 24% v/v acetonitrile, 0.1% v/v formic acid. The heated capillary temperature was set to 300° C and a static spray voltage of 2200 V was applied to the electrospray tip. The Fusion mass spectrometer was operated in the data-dependent mode, switching automatically between MS survey scans in the Orbitrap (AGC target value 400,000, resolution 60,000, and injection time of 50 milliseconds) with MS/MS spectra acquisition in the Orbitrap (AGC target value of 200,000, resolution 15,000 and injection time of 200 milliseconds) with quadrupole isolation. A three second cycle time was selected between master full scans in the Orbitrap mass analyzer; the ions were selected for fragmentation in the HCD cell with a normalized collision energy of 27%. Selected ions were dynamically excluded for 15 seconds with an exclusion mass width of ±10 ppm. Fusion data were analyzed using Proteome Discoverer 2.2 (Thermo Scientific) searching against a 2018 Uniprot human database that included common contaminants (29). A no-enzyme search was performed that used variable modifications of oxidation on methionine (+15.995 Daltons), phosphorylation on serine, threonine, tyrosine (+79.966 Daltons), cysteinyl on cysteine (+119.004 Daltons), deamidation on asparagine, and glutamine to pyroglutamic acid (-17.027 Daltons).

### MS Data Analysis

Data from both instruments were analyzed using the Sequest HT database search algorithm (30) and validated with Percolator (31). Resulting peptide lists were filtered to either a 1% or 5% FDR and culled of any peptides derived from source proteins listed within the CRAPome (32) or peptides having RPC retention times within 30 seconds of a longer, encompassing peptide. Venn overlap percentages comparing two datasets were calculated with the formula:

overlap percentage=((2×A2)/(A1+(2×A2)+A3))×100

where A1 is the number of peptides in set 1 but not in set 2, A2 is the number of peptides in common between sets 1 and 2, and A3 is the number of peptides in set 2 but not in set 1. We noted that calculating percent overlap in this standard manner tends to skew the percentage to lower values when the two datasets being compared were of very different sizes.

Peptide/allele binding predictions were made with NetMHCpan, version 4.1 (9), using the default settings on the web portal. Sequence logos were generated from culled, aligned MS peptide lists, showing the contribution to the relative entropy of a particular amino acid, symbolized by its single-letter code, to the distribution of amino acids at that position compared to the frequency distribution of amino acids in the UNIPROT database (32) as a reference. Amino acids are ranked at each position, with those deviating most from the reference frequency plotted tallest, and furthest from the x-axis. Positive entropy values indicate an amino acid that is enriched relative to reference, negative entropy values indicate an amino acid that is depleted relative to reference. The sum of all symbols (positive and negative) is the relative entropy in bits, compared to the reference. Logos were created in SVG using the Python package palmotif (https://github.com/agartland/palmotif/).

To compare two peptide sets A and B, we computed the KL divergence between the amino acid frequency distributions at each position in the peptide alignments. Convergence of the amino-acid distribution in A and B was expressed as a proportion of the KL divergence between A and B and a uniform distribution of amino acids. Convergence was also estimated as a function of the number of peptides in A (*N_A_*) from five to the total number of peptides observed; for each *N_A_* the peptides in A were subsampled without replacement 500 times to get an average convergence with peptides in B at the given *N_A_*. With relatively few peptides in A the convergence of A and B is low, however, as the number of peptides increases, the convergence increases at the positions which are similarly enriched in A and B. Convergence was also estimated for a peptide set with itself, as a function of the number of peptides observed. Though convergence was 1.0 by definition with all peptides, the rate of increasing convergence showed which positions were most enriched with specific amino acids.

All identified peptides, filtered by truncation and FDR criteria, across reported ARTEMIS experiments are included as **Supplementary Data**. Peptides from CRAPome-listed proteins are marked “sp”.

## Results

### Establishing the ARTEMIS Workflow

**Figure 1:**
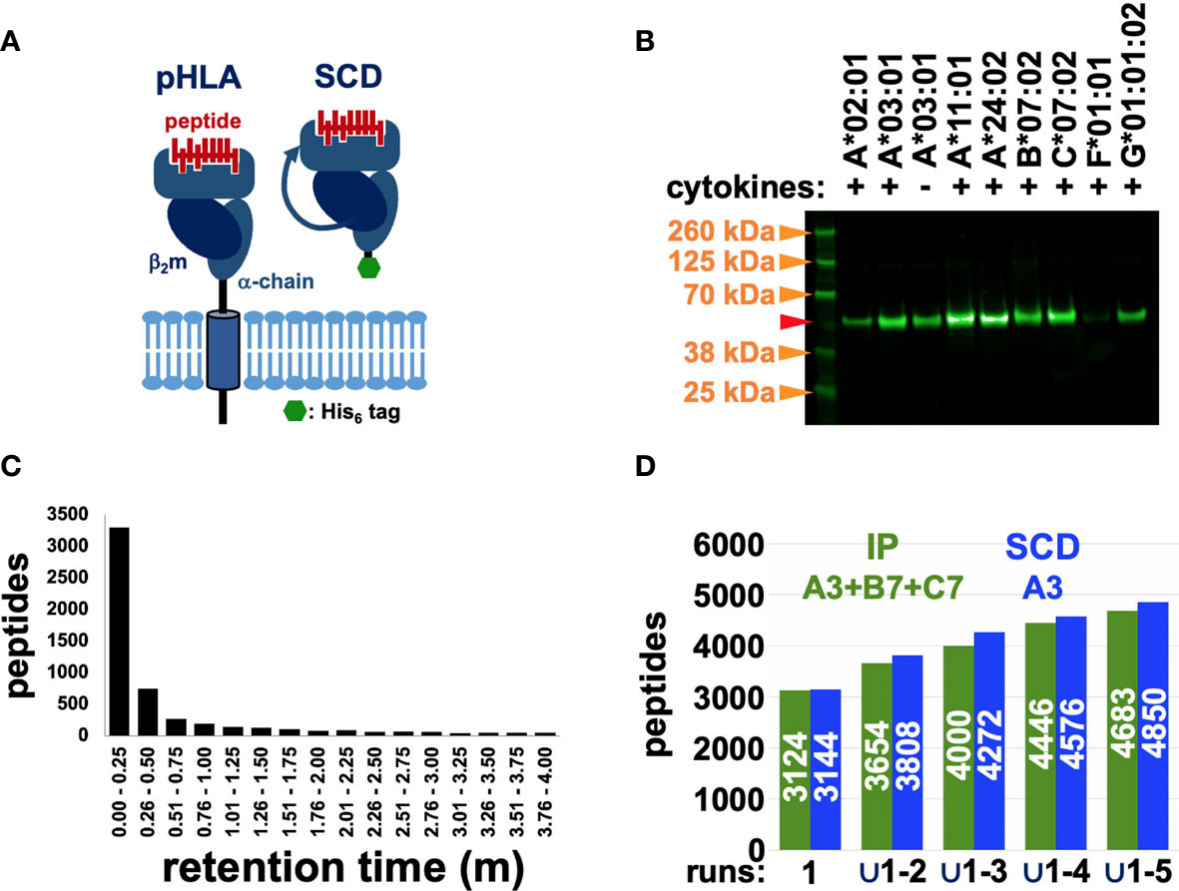
Using the SCD construct to recover and MS sequence HLA-restricted peptides. **(A)** Schematic representations highlight the differences between native, cell-surface pHLA and the engineered, secreted SCD construct. **(B)** Western blot analysis of SCD expression levels across alleles confirmed secretion of all tested SCDs, though at variable levels. Transduction, tissue culture, and Western conditions were closely matched across samples to allow meaningful comparisons of expression levels. The A3 SCD was tested with and without added cytokines (IFNγ, TNFα). Molecular weight markers (*orange arrows*) are indicated; the *red arrow* marks the expected PAGE mobility of an SCD with normal, expected N-glycosylation. **(C)** Chromatography retention times for nested peptides are graphed. The y-axis shows total number of unique peptide sequences in nested peptide sets in a co-elution time range; retention time intervals are indicated along the x-axis. **(D)** Accumulation rates of unique peptide sequences across five experimental replicates from the IP (*green*) and SCD (*blue*) datasets are compared (the pan-HLA IP dataset spans the three alleles in the haplotype of the cells analyzed, A3, B7, C7, but only A3 SCD results are shown). The y-axis shows total number of unique peptide sequences (also indicated in *white* in the bars) and progressively larger unions of five replicate datasets are indicated along the x-axis.

**Figure 2:**
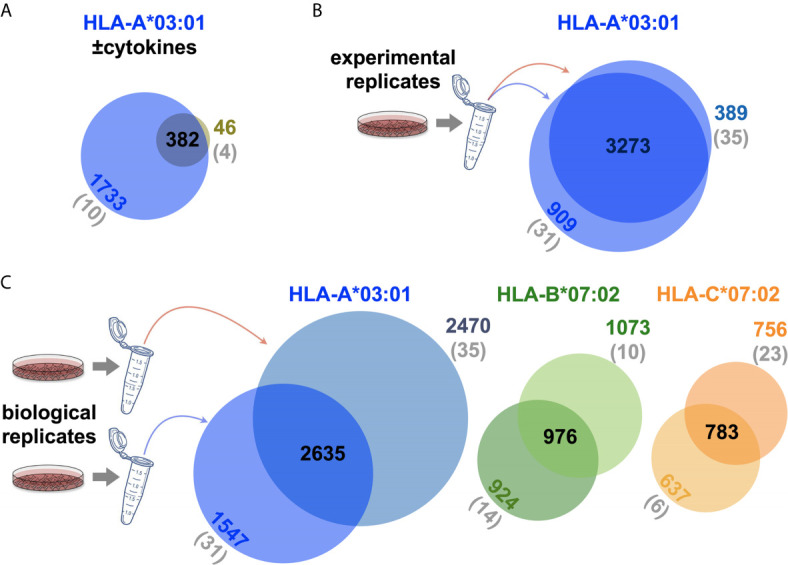
Intersections between SCD MS replicates. **(A)** Venn comparison of the unique peptides recovered by the A3 SCD transduced into cells cultured in the presence (*blue circle*) or absence (*green circle*) of cytokines to shift expression from conventional to immunoproteasomes. Numbers of peptides in the various intersection domains are indicated and the number of peptides that were culled because source proteins were listed in the MS CRAPome (29) are in parentheses, in *gray*. 89% of the cytokine-untreated peptides are observed in the cytokine-treated set. **(B)** Venn comparison of two A3 SCD experimental replicates are shown, labeled as in **(A)**. Overlap is 83%. **(C)** Venn comparison of two A3 (*shades of blue*), B7 (*shades of green*), and C7 (*shades of orange*) SCD biological replicates are shown, labeled as in **(A)**. Overlaps are 57% (A3), 49% (B7), and 53% (C7). Venn diagrams were generated with BioVenn (33).

We optimized HLA-I proteins as peptide-discovery reagents by truncating the transmembrane domain, generating a secreted form eliminating the need for detergent solubilization, appending a C-terminal poly-histidine purification tag to eliminate the need for antibody affinity chromatography, and linking the light and heavy chains into a single polypeptide (“*single chain dimer*” or SCD), stabilizing the protein and coordinating expression of both moieties (**Figure 1A**). SCDs were designed to retain the peptide binding specificity of native HLA-I proteins. SCDs were generated for seven HLA alleles and transduced into the human HEK293 cell line under carefully matched conditions. All were secreted into culture supernatants, validating the SCD design (**Figure 1B**). SCDs were purified from culture supernatants by immobilized metal affinity chromatography. Peptides were eluted from column-immobilized complexes with chaotropic agents, under reducing conditions to recover cysteine-containing peptides. Use of SCDs obviates the need for allele assignments and permits analyses independent of the source cell haplotype. Isolated peptides were fractionated by reversed-phase chromatography (RPC) and subjected to MS sequencing using Orbitrap instrumentation. Considerable effort was expended to optimize peptide purification and MS protocols, comparing gradient profiles and fractionation procedures, ion fragmentation methods, and choice of instrumentation. Many sets of “*nested*” (shorter peptides wholly contained within a longer identified sequence) RPC co-eluting peptides were observed, particularly using Fusion MS instrumentation, where shorter peptides were less binding motif-compliant than longer members in the nest, suggesting that in-source fragmentation was occurring during MS analysis (**Figure 1C**). To address this problem, shorter members of peptide nests were culled if they co-eluted within a narrow retention time window of 30 seconds. To assess the reliability and sensitivity of our final, optimized MS protocols, unique peptide sequence accumulation rates were compared across five “*technical*” or “*experimental*” replicates (replicate MS analyses of a single peptide eluate) for ARTEMIS, using the HLA-A*03:01 SCD, and the reported experimental replicates from the high-quality, conventional IP dataset (11) as a reference (**Figure 1D** and **Table 1**). Accumulation rates compared well, especially since the IP dataset spanned the three alleles in the haplotype of HEK293 isolate used (HLA-A*03:01, B*07:02, and C*07:02) and the ARTEMIS results were derived from a single SCD. MS-derived peptide lists were also filtered by MS false discovery rate (FDR) and culled by eliminating peptides from source proteins listed in the Contaminant Repository for Affinity Purification (“*CRAPome*”; e.g., **Figure 2**) (29).

**Table 1:** Averages of the run-by-run pairwise overlaps between peptides observed within the IP (A3+B7+C7) and SCD (A3) five-run union MS datasets.

	IP:	SCD:
**8-mers**	66.0%	45.8%
**9-mers**	76.4%	73.0%
**10-mers**	78.6%	74.2%
**11-mers**	79.0%	77.5%
**12-mers**	79.0%	77.5%
**13-mers**	77.7%	67.9%
**14-mers**	75.7%	73.1%
**8- to 14-mers**	76.1%	69.8%

The Western blot analysis (**Figure 1B**) had indicated that addition of proinflammatory cytokines (IFNγ, TNFα) during culture expansion after transduction increased SCD yield, consistent with known effects on proteasome subunit composition and activity (34). To examine this effect in more detail, ARTEMIS was performed with the HLA-A*03:01 SCD in the presence and absence of proinflammatory cytokines (INFγ and TNFα; **Figure 2A**), showing that the captured ligandome was dramatically expanded by more than four-fold with cytokine treatment, but not noticeably shifted: the intersection was considerably greater than observed with experimental (**Figure 2B**) or “*biological*” replicates (replicate MS analyses from separate SCD transductions, **Figure 2C**). Subsequent ARTEMIS analyses were therefore uniformly performed with cytokine treatment to expand peptide recovery. In order to compare experimental and biological replicates, Venn analyses were performed (**Figures 2B, C**), showing single-allele ligandome overlaps of ~80% in experimental replicates (consistent with **Figure 1** results), but dropping to ~50% in biological replicates.

### Validation of ARTEMIS SCD-Based, MS Peptide Identification

**Figure 3:**
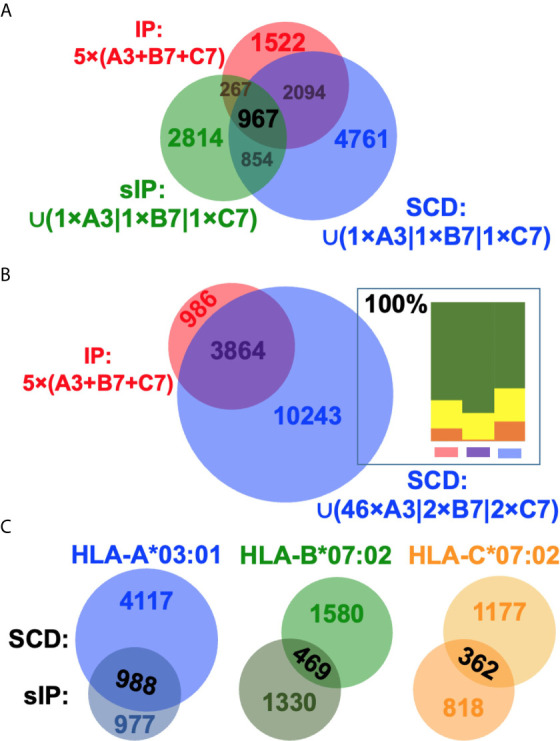
Comparing SCD, IP, and sIP MS dataset intersections. **(A)** Venn comparisons of the intersections of the IP five experimental replicate union dataset (*red*) and unions of three single-replicate sIP (*green*) or SCD (*blue*) single-allele (A3, B7, C7) datasets. Overlap is 45% between the IP and SCD datasets, corresponding to observation of 63% of the IP peptides in the SCD dataset. The overlap is 25% between IP and sIP datasets. **(B)** Venn comparisons of the intersections of the IP five experimental replicate union dataset (*red*) and the union of the available multi-replicate SCD (*blue*) single-allele (A3, B7, C7) datasets. 80% of the IP peptides were observed in the SCD replicate-union dataset. *Inset*: Peptides in each of the three Venn domains were analyzed for binding with NetMHCpan 4.1; results are plotted as relative percentage “strong binder” (*green*), “weak binder” (*yellow*), or “non-binder” (*orange*), summed over lengths and the three alleles in the analysis. **(C)** Venn comparisons of the intersections of A3, B7, or C7 sIP/SCD single-run datasets. Overlaps are 28% (A3), 24% (B7), and 27% (C7). Venn diagrams were generated with BioVenn (33).

**Figure 4:**
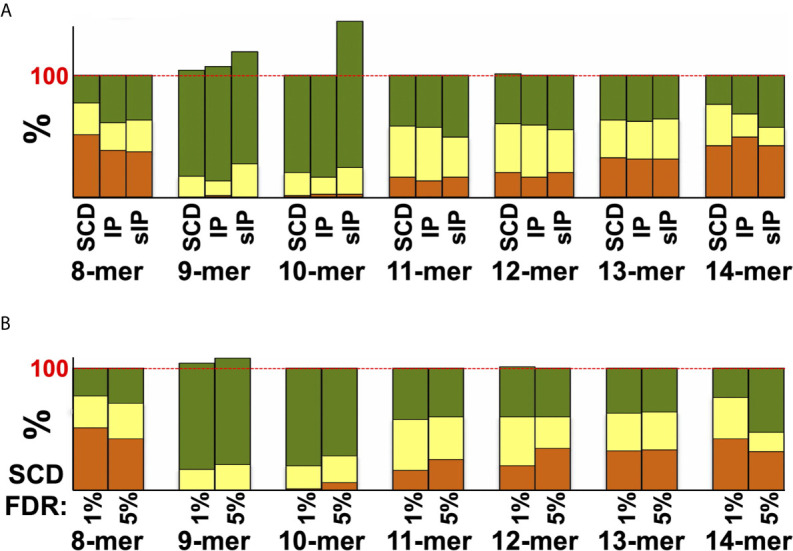
Agreement between MS datasets and NetMHCpan predictions. **(A)** Peptides from the pan-HLA IP A3+B7+C7 dataset, or the single-run sIP and SCD A3, B7, and C7 datasets used in prior comparisons, were analyzed by NetMHCpan for binding to these three alleles. Results are plotted as “strong binder” (*green*), “weak binder” (*yellow*), or “non-binder” (*orange*) and are binned by peptide length. **(B)** Single-run SCD A3, B7, and C7 datasets were analyzed by NetMHCpan and plotted as in **(A)**, comparing results from peptides identified at a 1% FDR cutoff or those added by expanding the cutoff to a 5% FDR. Results in **(A, B)** exceed 100% because NetMHCpan predicts that more peptides bind to multiple alleles than are observed in the MS datasets. In other words, many peptides were *predicted* to bind to more alleles in the set of three than were observed by MS, where most peptides were only identified binding to a single allele, contributing to overall binding scores of >100% when summed over all three alleles.

**Figure 5:**
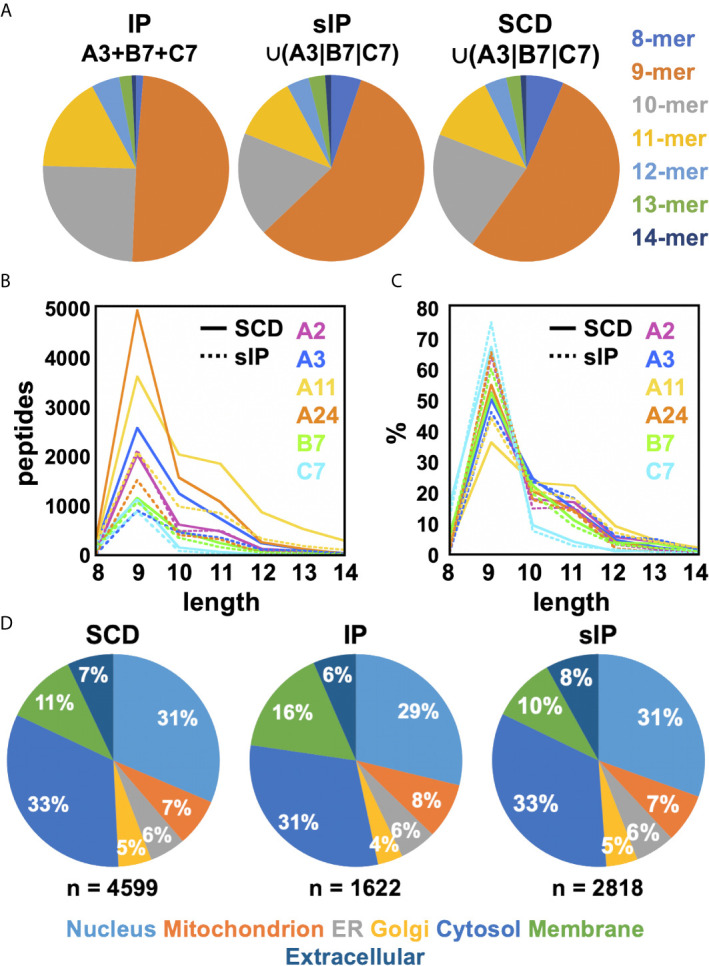
Comparisons of observed peptide length distributions recovered by MS. **(A)** The length distributions of peptides from the pan-HLA IP A3+B7+C7 dataset and the sIP and SCD union A3/B7/C7 datasets are displayed as pie charts. Peptide length distributions for single-run sIP and SCD analyses are compared, plotted as **(B)** absolute peptide numbers or **(C)** percentages. **(D)** Source protein subcellular localization profiles for the pan-A3/B7/C7 IP and union A3|B7|C7 sIP and SCD peptide datasets are shown as percentages. Compartments were labeled using Gene Ontology (GO) cellular compartment classifications for each peptide’s source protein: Nucleus [GO:0005634]; Mitochondrion [GO:0005739]; ER [GO:0005783]; Golgi [GO:0005794]; Cytosol [GO:0005829]; Membrane [GO:0016021]; or Extracellular [GO:0005576]/[GO:0005615] (36, 37).

Previous studies had argued that soluble versions of HLA-I proteins recapitulate native presentation (35) but to directly assess whether soluble SCDs accurately report HLA-I ligandomes, head-to-head comparisons were made with the high-quality IP and sIP reference datasets. In order to perform bias-free comparisons, the pan-HLA IP results, prior to clustering/deconvolution, which is comprised of peptides from across the cellular haplotype, were used in comparisons with union, “pseudo-pan” HLA-A*03:01/B*07:02/C*07:02 datasets constructed from combined single-allele sIP and ARTEMIS results, matching the haplotype of the cells used in the IP analysis. [Note that the IP results were the sum of five experimental replicates where the sIP pseudo-pan results were summed from three single runs.] Venn analyses of the joint, three-way overlap with an SCD dataset summed from three single runs (**Figure 3A**), or summed over available replicates (**Figure 3B**), showed comparable concordance, especially considering that these results are from three separate laboratories, using different MS protocols on two different cell lines, though also demonstrated that ARTEMIS tended to identify more total peptides. 967 peptides were observed in all three datasets. The observed 45% overlap between IP and summed, single-run SCD datasets approached the ~50% overlap observed between SCD biological replicates, corresponding to recovery of 63% of the IP peptides using SCD reagents. This recovery rate rose to 80% (3864 out of 4850 total peptides) combining available SCD replicates (**Figure 3B**). Analysis of NetMHCpan predicted binding quality (strong/weak/non-binding) did not show dramatic differences between the Venn overlapping peptides and the IP-only and SCD-only peptides (**Figure 3B**, *inset*). Venn analyses comparing sIP and ARTEMIS results allele-by-allele (**Figure 3C**) showed overlaps of ~25%. SCD, IP, and sIP results were also analyzed with NetMHCpan (**Figure 4A**) to assess compliance with predicted binding motifs, which also showed consistent agreement across the three MS methods. We also analyzed ARTEMIS peptides identified at a 1% FDR cutoff with peptides added by expanding to a 5% cutoff (**Figure 4B**), which showed that agreement with prediction was consistent even with more relaxed inclusion criteria, suggesting that these additional peptides may be valid binders. We therefore report ARTEMIS results including peptides identified at a 5% FDR, flagged as such (**Supplementary Data**). Observed 8- to 14-mer peptide length distributions were also consistent across the pan-HLA IP and pseudo-pan sIP and ARTEMIS union datasets (**Figure 5A**) and the sIP and ARTEMIS results on an allele-by-allele basis (**Figures 5B, C**). Source protein subcellular localization profiles were also in very close agreement across IP, sIP, and SCD identified peptides, with between 6% and 8% of observed peptides derived from extracellular proteins (**Figure 5D**).

### ARTEMIS Estimation of HEK293 Allele-Specific Presentomes

**Figure 6:**
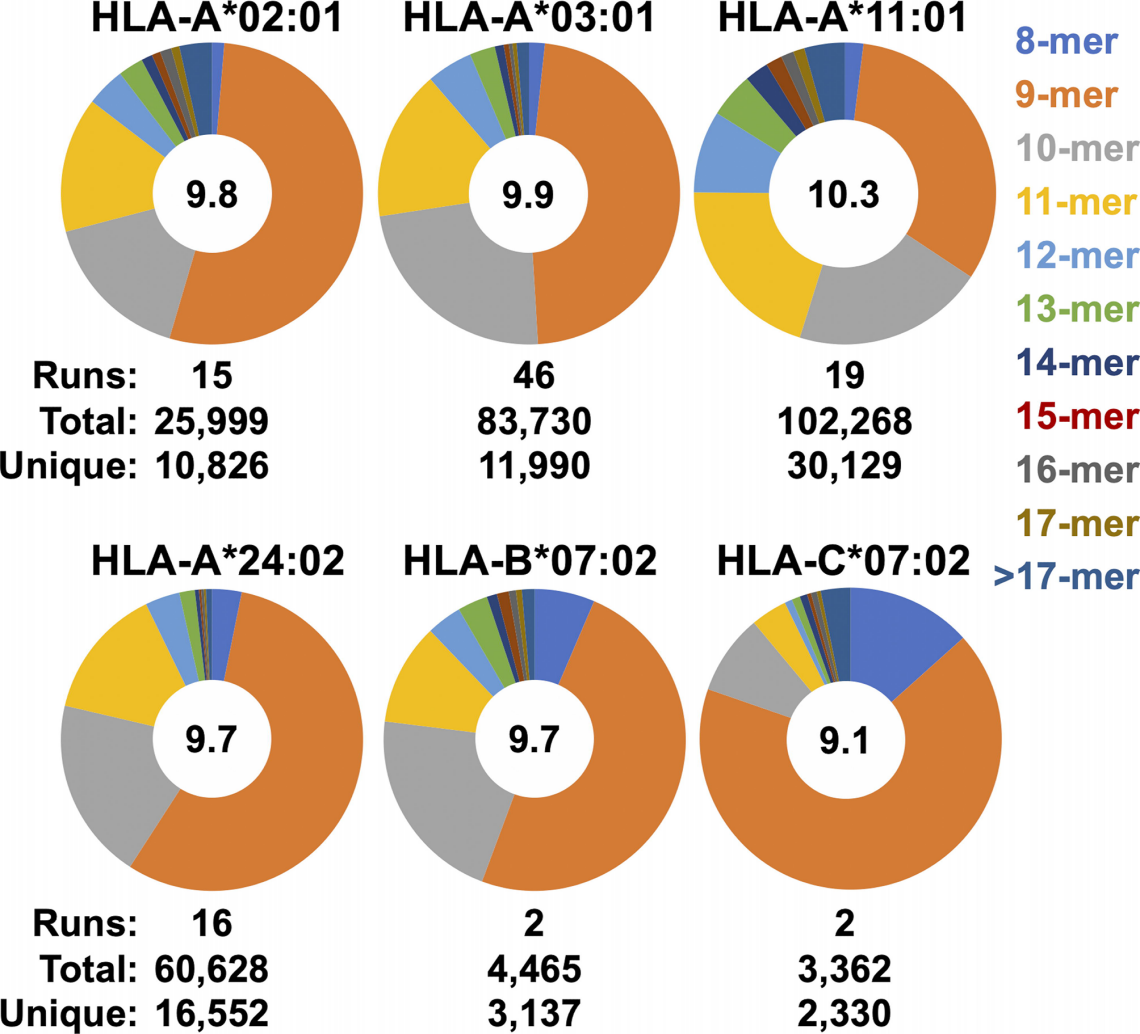
Observed peptide length distributions recovered from SCDs by allele. The length distributions of peptides from merged SCD analyses across multiple replicates are displayed as pie charts. SCD allele and peptide length are shown as indicated. Numbers of replicates (“runs”), total peptides observed, and the number of unique peptides in the union datasets are indicated. Average peptide length across the merged datasets is shown in the in the center of each pie.

The ease with which ARTEMIS can be performed, and the extensive optimization studies we performed, led to the accumulation of an enormous amount of MS data, particularly for the HLA-A alleles we studied (**Figure 6**). With more than a dozen replicate runs performed, accumulation of unique peptide sequences in cross-run, HLA-A union datasets tended to converge, suggesting that these unions represented good estimates of the limiting, allele-specific presentomes from HEK293 cells. The total breadth of these presentomes varied across alleles, with HLA-A*02:01 having the most limited presentome and HLA-A*11:01 having the most expansive. [HLA-B*07:02 and HLA-C*07:02 are not included in this comparison as insufficient replicates were performed to achieve convergence.] Allele-specific peptide length distributions calculated across these replicates also revealed differing length preferences, with HLA-A*11:01 skewing to longer peptides and HLA-C*07:02 skewing to shorter peptides. The observed average presented peptide length ranged from just greater than nine to greater than ten residues. [HLA-B*15:01 is not included in this comparison as only one run has been performed so far].

### ARTEMIS-Derived, Length-Specific, Peptide Sequence Motifs

**Figure 7:**
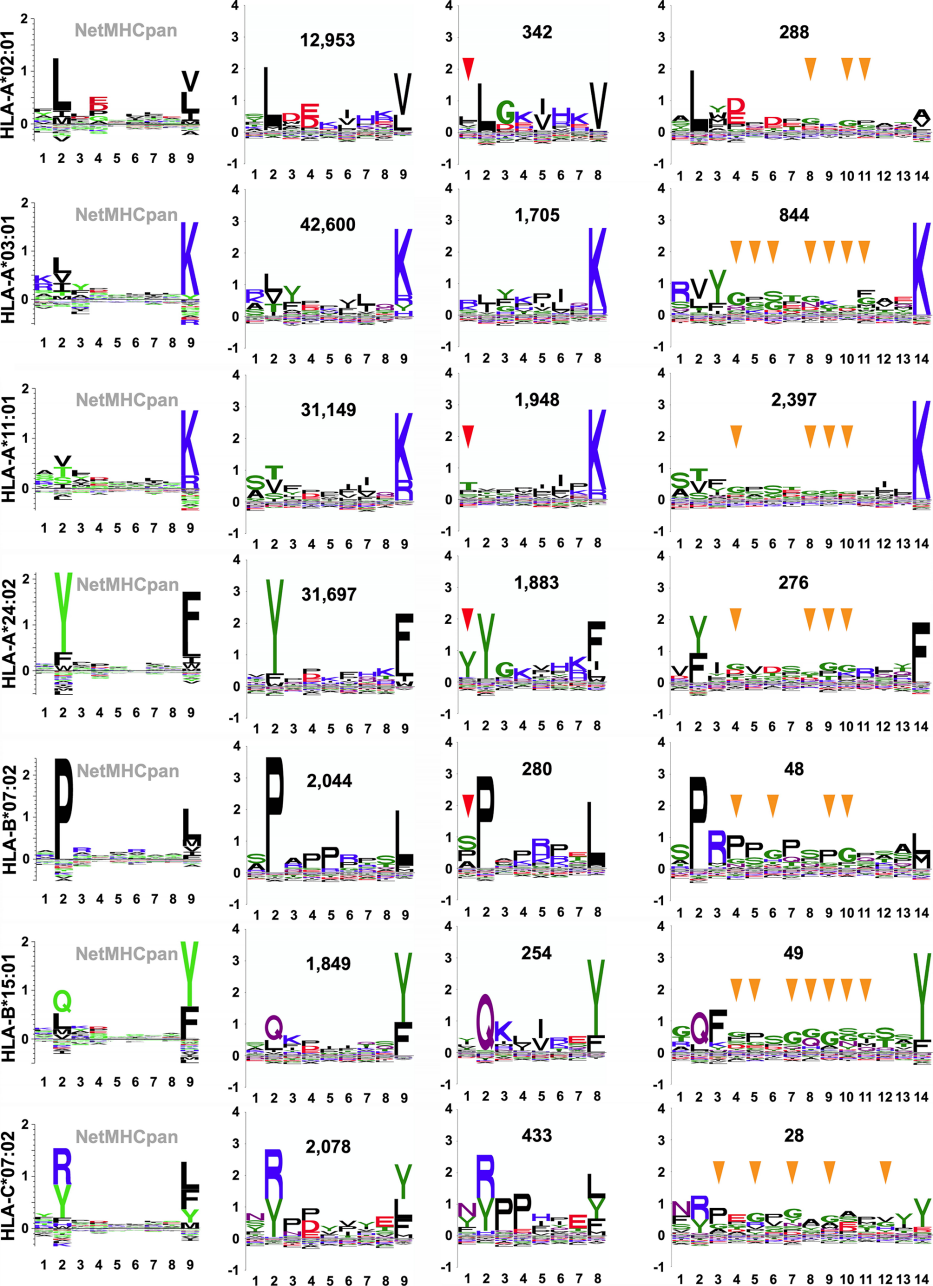
SCD-derived, allele- and length-specific peptide sequence logos. Allele-specific recognition motifs are presented as sequence logos, either as reference 9-mer logos from “*naturally presented ligand*” peptides available through the NetMHCpan motif viewer portal (*column 1*; http://www.cbs.dtu.dk/services/NetMHCpan/logos_ps.php) or as custom logos, generated as part of this work, from SCD-recovered peptides (*columns 2 through 4*). The x-axis reports position in the peptide, the y-axis reports information content of different residues at that position, in bits. Alleles are specified at *left*, and the total number of SCD-recovered peptides used to generate that logo is inset (*columns 2 through 4*). For SCD-recovered peptides, only logos generated from 8-mers, 9-mers, and 14-mers have been selected for display for simplicity. P1 positions that echo the P2 anchor position amino acid preference in 8-mer logos are indicated by *red arrows* and positions in 14-mer logos where glycine rises in abundance relative to 9-mers are indicated by *yellow arrows*.

We constructed customized, length-specific sequence logos from ARTEMIS results, weighting information content by the observed frequency distribution of amino acids in human proteins (**Figure 7**). ARTEMIS results were completely consistent with available reference logos, further validating the accuracy of SCD-based methods, but also revealed informative nuances when logos separately calculated for different lengths were compared. Auxiliary anchor residue identity and strength shifted across lengths. Motifs calculated from 8-mers showed the P2 anchor residue preference partially shifting to the P1 position for several alleles, particularly HLA-A*24:02 (**Figure 7**), but never with the complete loss of the usual P2 anchor preference. 14-mer logos calculated from ARTEMIS results also showed variations from 9-mer logos, with glycine residues rising in abundance in the middle of these longer peptides.

### Evaluation of HLA-I Supertype Overlaps

**Figure 8:**
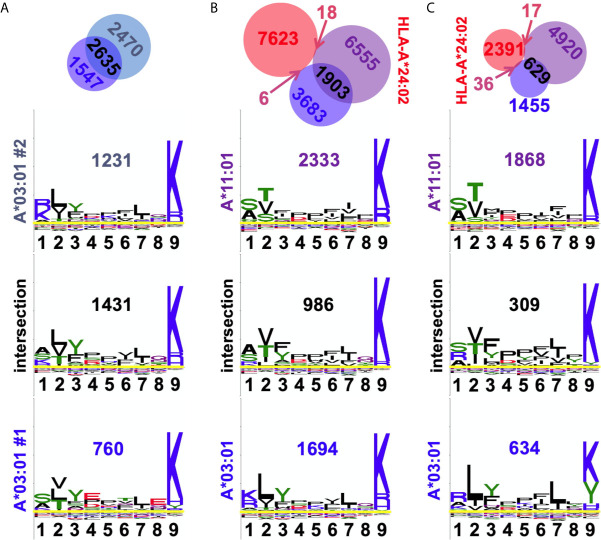
Observed peptide overlaps in the A3/A11 HLA supertype. **(A)** The A3 biological replicate intersection is shown at *top*, echoing **Figure 3C**. *Below* are shown the 9-mer sequence logos for the residues in the three Venn domains, with numbers of peptides inset. The x-axis reports position in the peptide, the y-axis is scaled to the information content of different residues at that position. Overlap is 57%. **(B)** Venn analyses of SCD A3 (*blue*), A11 (*purple*), and A24 (*red*) results are shown with the numbers of peptides in each domain indicated. *Below* are shown the 9-mer sequence logos for the residues in the three SCD Venn domains, with numbers of peptides inset. The overlap between SCD A3 and A11 datasets is 27%. **(C)** Venn analyses of sIP A3 (*blue*), A11 (*purple*), and A24 (*red*) results are shown with the numbers of peptides in each domain indicated. [A24 results are shown as an orthogonal comparator.] *Below* are shown the 9-mer sequence logos for the residues in the three sIP Venn domains, with numbers of peptides inset. The overlap between sIP A3 and A11 datasets 16%. Venn diagrams were generated with BioVenn (33).

**Figure 9:**
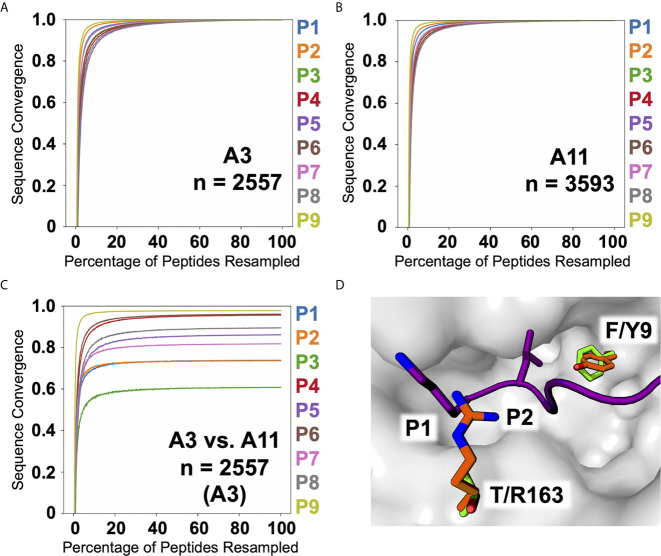
A3, A11, and A3 versus A11 KL sequence divergence. KL divergence between the amino acid frequency distributions at each position in the peptide alignments is plotted as sequence convergence (*y-axis*) versus percentage of peptides resampled (*x-axis*) for the SCD A3 9-mer dataset against itself **(A)**, the SCD A11 9-mer dataset against itself **(B)**, and the SCD A3 9-mer dataset against the SCD A11 9-mer dataset **(C)**. Individual peptide position-by-position convergences are colored as indicated along the *right*. **(D)** A structural view highlights sequence differences between A3 and A11 affecting P2 amino acid preference. The molecular surface of A3 (2XPG.pdb; *grey*) is shown with the backbone of the bound peptide shown as a cartoon ribbon (*purple*) with the P1 and P2 side-chains shown in a licorice-stick representation.

Two of the alleles we studied, HLA-A*03:01 and A*11:01, fall within the same HLA supertype (19, 20), predicting conserved peptide recognition. Venn analyses of ARTEMIS and sIP results (**Figures 8A–C**) showed overlaps between these two alleles (27 or 16%; **Figures 8B, C**) higher than overlaps with alleles with orthogonal specificities, but less than typical for biological replicates from the same allele (~50%; **Figure 8A**). In order to more finely parse supertype specificities, logos were generated from the three Venn domains, the two non-overlapping peptide sets and the intersection set. While 9-mer logos from these three Venn domains from a pair of HLA-A*03:01 biological replicates showed very similar logos (**Figure 8A**), strongly matching at the P2 and P9 anchor positions, HLA-A*03:01 and A*11:01 overlap 9-mer logos showed segregation of peptides, particularly at the P2 anchor position, comparably in both the ARTEMIS and sIP datasets (**Figures 8B, C**). To quantify this effect, Kullback–Leibler (KL) divergence (38) was calculated to estimate whether an adequate number of peptide sequences had been observed to converge on a defined recognition motif and if comparisons of HLA-A*03:01 and A*11:01 peptides converged to a single recognition motif. Using the SCD A*03:01 and A*11:01 9-mer datasets as examples (**Figures 9A, B**), motifs converged with about 1500 peptide sequences, but comparisons of A*03:01 and A*11:01 unions failed to achieve KL convergence to a single recognition motif, particularly at the P1, P2, and P3 positions, confirming recognition divergence (**Figure 9C**). Sequence differences between HLA-A*03:01 and A*11:01 at positions bracketing the P2 pocket provide a reasonable structural explanation for the observed sub-specificity differences (**Figure 9D**).

### Using ARTEMIS to Identify Peptides From Co-Transduced Target Proteins-of-Interest

**Figure 10:**
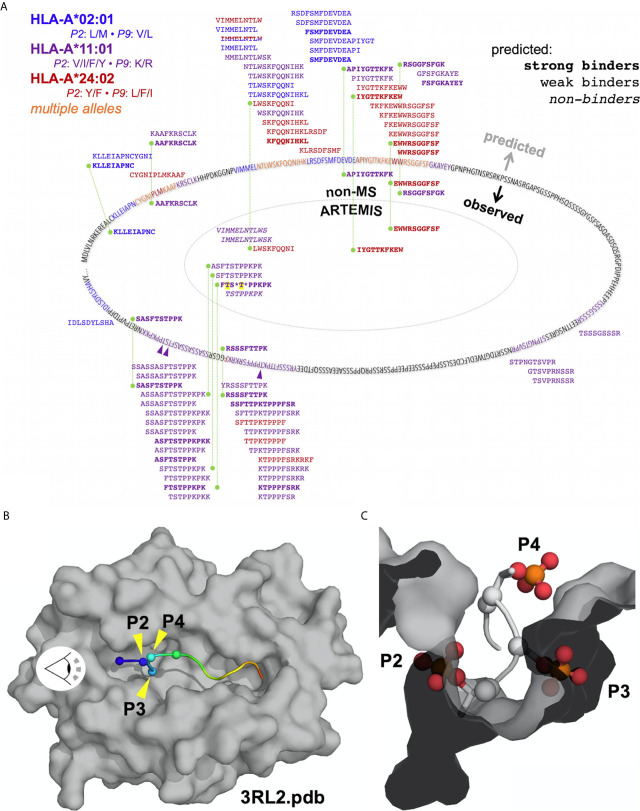
Mapping ARTEMIS-identified peptides onto the LT sequence. **(A)** The sequence of the truncated form of MCV LT associated with cancer is shown mapped onto an oval. Residues in ARTEMIS-identified peptides are colored as indicated; phosphorylated threonine residues are marked with *purple arrows*. LT peptides predicted by NetMHCpan to bind to A2, A11, or A24 are shown around the outside of the oval, approximately positioned by their location in the sequence. Predicted strong binders are shown in *bold*, peptides predicted not to bind are shown in *italics*. Peptides identified by non-MS experimental methods are shown inside the sequence oval but outside the *dotted line*, colored by allele as indicated. All of these peptides are predicted to bind strongly to their cognate alleles. ARTEMIS-identified peptides are shown inside the *dotted line*, colored by cognate allele as indicated, with predicted binding/non-binding behavior shown in *bold* or *italics*. Phosphorylated residues in ARTEMIS-identified peptides are indicated by *asterisks*; threonines phosphorylated in LT in ARTEMIS-identified peptides are highlighted in *yellow*. *Green dashed lines* connect peptides identified by more than one method. **(B)** The crystal structure of A11 PDB accession code 3RL2 (51) is shown as a molecular surface, colored *gray*, with the backbone of the bound peptide shown in a cartoon representation, colored from *blue* to *red*, N- to C-terminus. The α-carbons of the first four residues in the peptide (P1 through P4) are marked with *spheres*. The eye symbol indicates the viewpoint shown in **(C)**. **(C)** A view down the peptide binding groove of A11 from the perspective indicated in **(B)**. Phosphothreonine residues have been modeled into the P2 and P4 positions, and a phosphoserine has been modeled into the P3 position. Phosphate groups have been shown in a ball-and-stick representation, colored by atom type.

**Figure 11:**
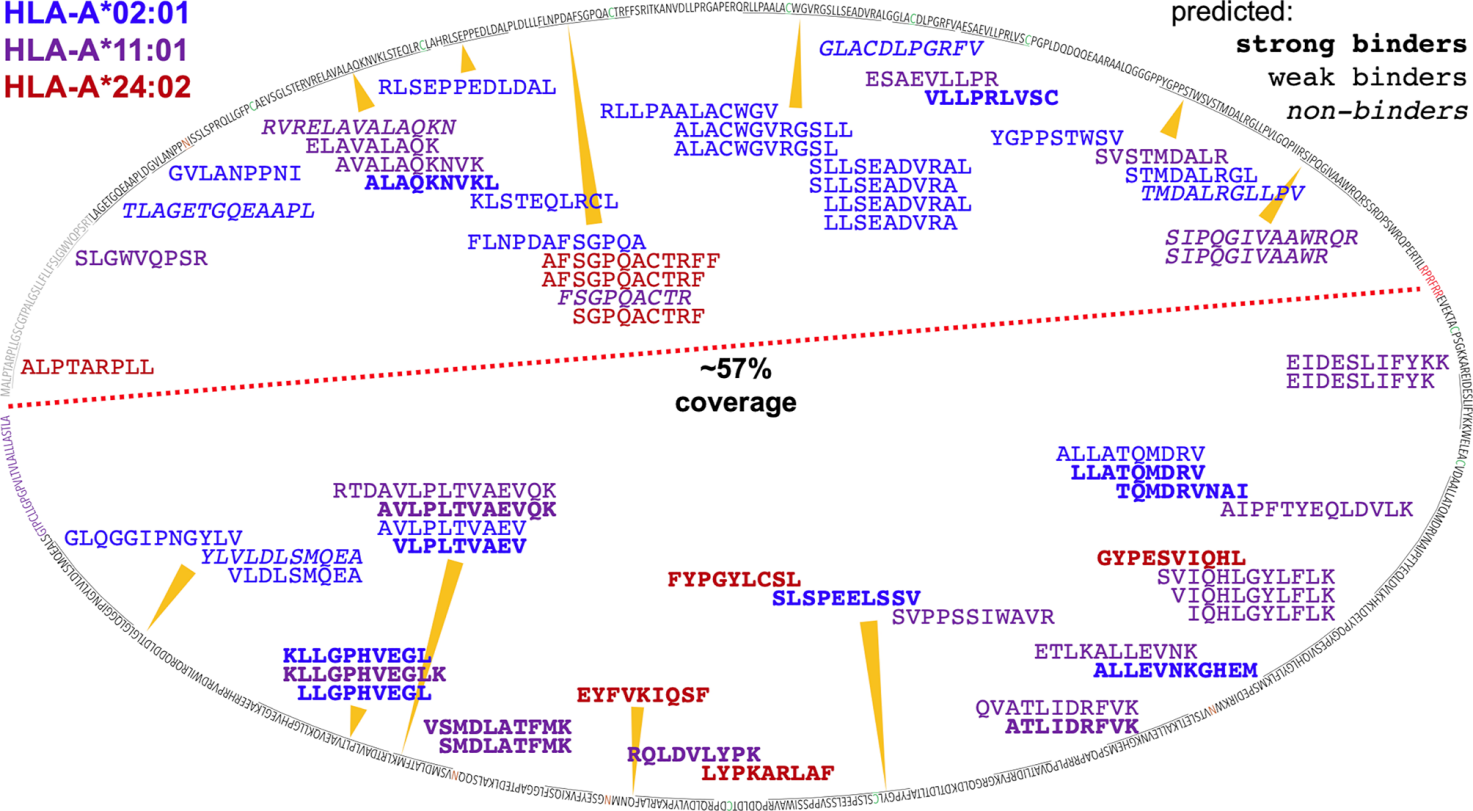
Mapping ARTEMIS-identified peptides onto the MSLN sequence. The sequence of MSLN is shown mapped onto an oval. The leader peptide is colored *gray*, the furin cleavage site is colored *red*, the 4x potential N-glycan sites are colored *orange*, and the GPI signal sequence is colored *purple*. The *red dashed line* separates the MSLN proprotein sequence into MPF and MSLN moieties. ARTEMIS-identified peptides are shown inside the oval, approximately positioned by their location in the sequence, colored by cognate allele as indicated, with predicted binding/non-binding behavior by NetMHCpan shown in *bold* or *italics* as indicated. 57% of the MPF/MSLN sequence is presented as A2, A11, or A24 peptides identifiable *via* ARTEMIS. The MPF moiety of the MSLN fusion precursor proteins is highly expressed as a secreted protein *via* transduction in HEK293 cells, ten-fold or more higher in culture supernatants than SCDs, so may contaminate the isolated SCDs. However, observed coverage was comparable over the two moieties, including peptides from the leader sequence (which are not derived from a secreted contaminant but from endogenously expressed MPF/MSLN), suggesting that contamination was not a huge issue. 115 A2, A11, and A24 peptides were predicted to bind, using NetMHCpan, that were not observed by ARTEMIS. These peptides have not been shown to avoid excessive clutter.

ARTEMIS reported thousands-deep, allele-specific ligandomes from cells transduced with the SCD reagent. Use of optimized lentiviral constructs can yield transduction efficiencies approaching 100% (22), enabling efficient co-transductions with more than one lentivirus construct. We leveraged this property in ARTEMIS by co-transducing HLA-A*02:01, A*11:01, or A*24:02 SCDs with a lentivirus encoding a target protein, yielding a thousands-deep, allele-specific ligandome from HEK293 cells plus peptides from the targeted proteins: E6 and E7 from HPV16, an HIV Env gp140, the truncated, tumor-associated form of MCV LT, or the tumor-associated antigen MSLN in its native, proprotein form (**Table 2**, **Figures 10**, **11**). A small number of these peptides had been identified or evaluated by some previous experimental approach (**Table 2**), but many were novel. These HLA-restricted peptides represent potential therapeutic targets but also began to reveal fundamental aspects of peptide processing and presentation. We generated NetMHCpan binding property predictions for all ARTEMIS-identified peptides from target proteins (**Table 2**) and used NetMHCpan to predict all 8- to 14-mers from LT and MSLN presented by the three A alleles tested as SCDs (**Figures 10**, **11**). For LT, we compared NetMHCpan predictions with peptides identified by non-MS experimental methods [recently and thoroughly compiled (52)] and ARTEMIS-identified peptides, mapping them onto the LT sequence (**Figure 10**). ARTEMIS-identified peptides from MSLN were also mapped onto its sequence (**Figure 11**). Consistent with the ligandome results tabulated in **Figure 4** across MS techniques, ARTEMIS results from these targeted proteins include many peptides predicted not to bind (prediction false negatives) and NetMHCpan predicts many peptides that were not observed (prediction false positives).

**Table 2:** Peptides from target proteins recovered from SCD/target protein co-transductions.

	HPV 16 E6/E7	MCV LT	MPF/MSLN	HIV ENV (SF162)
HLA-A*02:01	**TIHDIILEC** **TIHDIILECV** YMLDLQPETTDL	—	**ALAQKNVKL** ALLEVNKGHEM KLLGPHVEGL LLATQMDRV LLGPHVEGL RLSEPPEDL SLSPEELSSV TQMDRVNAI VLLPRLVSC **VLPLTVAEV** ALLATQMDRV AVLPLTVAEV FLNPDAFSGPQA GLQGGIPNGYLV GVLANPPNI KLSTEQLRCL **LLSEADVRA** LLSEADVRAL RLLPAALACWGV *RLSEPPEDLDAL* **SLLSEADVRA** SLLSEADVRAL STMDALRGL VLDLSMQEA **YGPPSTWSV** YLVLDLSMQEA *ALACWGVRGSL* *ALACWGVRGSLL* *GLACDLPGRFV* SLLSEADVRALGGL *TLAGETGQEAAPL* *TMDALRGLLPV*	—
HLA-A*11:01	**GTTLEQQYNK** SVYGTTLEQQY SVYGTTLEQQYNK **TTLEQQYNK** AVCDKCLKFYSK **AMFQDPQER** **AVCDKCLKFY**	ASFTSTPPKPK FTS*T*PPKPK IMMELNTLWSK TSTPPKPK VIMMELNTLWSK	ATLIDRFVK **AVLPLTVAEVQK** FTYEQLDVLK KLLGPHVEGLK RQLDVLYPK SMDLATFMK VSMDLATFMK AVALAQKNVK EIDESLIFYK EIDESLIFYKK ELAVALAQK ESAEVLLPR ETLKALLEVNK IQHLGYLFLK QVATLIDRFVK RTDAVLPLTVAEVQK RVNAIPFTYEQLDVLK SLGWVQPSR SVIQHLGYLFLK SVPPSSIWAVR SVSTMDALR VIQHLGYLFLK AIPFTYEQLDVLK *FSGPQACTR* RVRELAVALAQKN *SIPQGIVAAWR* *SIPQGIVAAWRQR*	AISSVVQSEK AVFVSPSASVEK ISSVVQSEK NTLKQIVTK **VTVYYGVPVWK** ASLWNWFDISK GTITLPCRIK WGIKQLQAR
HLA-A*24:02	**PYAVCDKCLKF** **VYDFAFRDL** **VYCKQQLL** VCDKCLKF **VYDFAFRDLCI**	**EWWRSGGFSF** IYGTTKFKEW LWSKFQQNI	*EYFVKIQSF* *FYPGYLCSL* *GYPESVIQHL* *LYPKARLAF* *AFSGPQACTRF* *AFSGPQACTRFF* ALPTARPLL SGPQACTRF YPESVIQHL	AYDTEVHNVW KMQKEYALF KWASLWNWF LYKYKVVKI MYAPPIRGQI NWFDISKWLW **RYLKDQQLL** VWKEATTTL VWKEATTTLF VYYGVPVWKEATTTLF NYTNLIYTLI RYLKDQQL

NetMHCpan-predicted binding: strong binder, dark green; weak binder, light green; and non-binder, black. Peptides shown in bold have previously been reported on the basis of some experimental approach (17, 39–50). MSLN peptides in italics are from the MPF moiety, which is highly expressed as a secreted protein via transduction in HEK293 cells, so may contaminate the isolated SCD. Peptides are listed in alphabetical order within groups. Asterisks indicate phosphorylated residues in one LT peptide; the serine is phosphorylated in 7.4% of observations, the threonine is phosphorylated in 92.6%.

## Discussion

Applying multiple criteria over a series of comparisons, ARTEMIS SCD-based peptide identifications concorded with those reported from conventional, immunoprecipitation-based MS methods and reference binding motifs, validating that ARTEMIS accurately reports HLA-I ligandomes, in terms of specific peptides identified, reported binding motifs, and length distributions, while adding additional, informative nuance to these results (e.g., allelic variations in average bound peptide length and limiting presentome sizes). Reliability was increased through the conservative filtering of potentially false-positive results, though many of the CRAPome-derived and higher FDR peptides (**Figure 4B**) were motif-compliant, so our applied rejection criteria may be overly conservative. One advantage of ARTEMIS is the simplicity of the workflow, which readily enabled full biological replicates in high multiples to be performed. The decreased overlaps observed for biological replicates relative to experimental replicates reasonably reflects unavoidable experimental variation and stochasticity, but likely also the dynamic nature of HLA ligandomes. This is an important consideration for assessing reproducibility but also provides the means to analyze ligandome dynamism in the future.

However, a disadvantage of ARTEMIS is the dependence on lentiviral transduction, which likely precludes analyses of primary cells and other slowly dividing cell types. MS analyses performed in cell lines, like ARTEMIS, also report ligandomes that are unlikely to recapitulate natural contexts, like heterogenous solid tumors – or even different cell lines – because of the myriad biological factors that affect peptide processsing and presentation. Peptides identified by any technique also need to be independently validated for binding and presentation in a physiological context prior to clinical exploitation. MS-based identifications inherently cannot determine conclusively that an unobserved peptide is not presented. For these reasons, we advocate for high-replicate MS analyses of ligandomes which achieve convergence to improve identification confidence.

Use of soluble HLA-I reagents also raises several potential, theoretical concerns because these reagents would not be expected to interact natively with the intracellular peptide loading and editing machinery. These concerns include the ability to efficiently fold and secrete soluble HLA-I molecules in the absence of interactions with chaperones, the ability to efficiently present swapped-in higher affinity peptides, and to present peptides derived from proteins across cellular compartments, particularly extracellular and secreted proteins. However, our preliminary results allayed these concerns. SCDs are efficiently secreted from cells, passing through secretion pathway quality-control checkpoints (**Figure 1B**). Ratios of strong/weak/non-binding peptides observed, as defined by NetMHCpan predictions, were concordant across MS techniques (**Figure 4A**). Sub-cellular compartment distributions of peptide source proteins, particularly extracellular proteins, were also concordant across techniques (**Figure 5D**). We also noted efficient, even recovery of peptides across MSLN fusion protein precursor domains, including both secreted (MPF) and extracellular cell-surface (MSLN proper) moieties. There is also the potential concern that the HLA-I proteins comprising the endogenous haplotype might out-compete haplotype-matching SCDs for peptides, affecting observed repertoires. However, this concern was allayed by comparison of ARTEMIS results in HEK293 cells with HLA-A*03:01, -B*07:02, and -C*07:02 SCDs matching the HEK293 HLA haplotype of the conventional IP reference dataset and overlapping the haplotype of our HEK293 isolate (HLA-A*02:01, -03:01, -B*07:02, and -C*07:02; **Figure 3B**). Using summed, single-replicate ARTEMIS results, the observed overlap (45%) corresponded to recovery of 63% of the peptides in the IP dataset, rising to 80% recovery using summed, multi-replicate ARTEMIS results, showing efficient sampling of the native HLA-I ligandome even when native HLA-I alleles matching the SCD used were present in the cell line’s haplotype.

These analyses also highlighted caveats with computational prediction. While 9-mers observed by IP, sIP, and ARTEMIS MS approaches showed almost complete agreement with predicted binding, the percentage of MS observed peptides that would have been *predicted* not to bind increases dramatically as the length deviates from 9-mers consistently across all three MS methods (**Figure 4A**), demonstrating high false negativity relative to MS-based experimental approaches. NetMHCpan also tended to overpredict binding of a peptide to multiple alleles, and peptides identified from co-transduced target proteins (**Table 2**), leading to increased false positivity.

Sequence variations in observed ARTEMIS-derived, length-specific logos can be correlated with known variations in peptide binding, increasing confidence in ARTEMIS results. Numerous crystal structures of pHLA complexes reveal the binding mechanisms of HLA-I proteins, which have a binding groove optimally sized to bind 9- or 10-mer peptides (53). Two different binding modes have been observed in 8-mer pHLA structures: 8-mers typically stretch out to fully occupy the too-long groove, but at least one 8-mer pHLA structure [PDB accession code 1DUY (54)] showed an alternate mode, where the peptide incompletely filled the groove, with the peptide P1 side-chain occupying the P2 specificity pocket of the HLA protein, leaving the N-terminus pocket empty. This binding mode has implications for observed motifs: the P2 anchor residue preference should shift to the P1 position of the peptide. In ARTEMIS results, this behavior was observed for several alleles (**Figure 7**), particularly HLA-A*24:02, but never to the exclusion of the typical binding mode, suggesting that this alternate 8-mer binding mode is frequently employed, but unevenly over peptides and alleles. This interpretation needs to be confirmed by 8-mer pHLA structural studies, partly to ensure that this effect is not due to contamination with artificially truncated peptides, despite our careful filtering algorithms, and partly to determine sequence/structural motifs determining binding mode selection. Structural studies show that longer peptides tend to bind with termini anchored in the N- and C-terminus pockets in the HLA groove but with additional residues extruding out of the middle of the pocket, with obvious implications for TCR recognition, e.g. (55), ARTEMIS results from 14-mers, with glycine residues rising in abundance in the middle of these longer peptides, was consistent with enabling the flexibility needed to accommodate extrusion.

HLA supertypes potentially complicate conventional IP MS analyses, which require clustering prior to allele assignment: if the haplotype is comprised of alleles within a supertype, the number of clusters to select may not be clear. Analysis of ARTEMIS results from HLA-A*03:01 and A*11:01 (**Figure 8**) showed potentially the worst possible outcome for cluster number selection: though obviously overlapping, the specificities of these two alleles from within the same supertype were distinct enough to clearly segregate subsets of peptides. It was not clear from our analysis how to define, *ab initio*, cluster number to capture this nuance adequately.

Sequence differences between HLA-A*03:01 and A*11:01 around the P2 position provide a reasonable structural explanation for the observed sub-specificity differences (**Figure 9D**) which may affect other members of other HLA supertypes. There are four amino acid positions in the peptide binding cleft, which make contact with bound peptide, that differ between A3 and A11: F9Y, E152A, L156Q, T163R. Residues at positions 152 and 156 contribute hydrophobic interactions to the E and D pockets, respectively, and accommodate similar anchor residues across the two alleles. Residues at positions 9 and 163, however, add constraints on which amino acids are preferred in the A and B pockets, respectively. At position P2, A3 preferred L or I, and A11 preferred T or S, due to the presence of either a tyrosine at position 9 in A11 or a phenylalanine in A3. The smaller phenylalanine side-chain allows for the branched hydrophobic residues seen in A3 presented peptides, whereas the larger and more polar Y dictates that P2 in A11 presented peptides have smaller and more polar residues.

Analyses of KL convergence of sequences also defined the number of peptide observations needed to determine a converged, and presumably reliable, motif logo and further validated ARTEMIS results by showing that single-SCD results converged on a single motif.

MCV LT provided an excellent opportunity to compare different peptide discovery methods (**Figure 10**) with a recent study performing rigorous ELISPOT assays and compiling previous results across non-MS techniques (52). We performed ARTEMIS analyses and NetMHCpan predictions with three SCDs (HLA-A*02:01, A*11:01, and A*24:02; **Figure 10**) to complement these results. Predicted and experimentally observed peptides very unevenly sampled the LT sequence, which contains two large, fairly low sequence complexity blocks likely accounting for the uneven distribution. All experimental approaches returned far fewer peptides than prediction, again highlighting high false-positive rates. All T cell assay-based identifications were predicted to be strong HLA binders, though prediction algorithms are often used to delineate the presented peptide within longer synthetic sequences used in the assays. ARTEMIS peptides, consistent with bulk ligandome analyses, identified peptides predicted to be a mix of strong, weak, or non-binders. Prediction, T cell assays, and ARTEMIS all identified the A*24:02-restricted 10-mer EWWRSGGFSF, which is perhaps the best characterized and validated HLA-restricted LT epitope. However, there were no other identical matches between the non-MS and ARTEMIS experimental results, though several overlapping peptides were differentially identified. We point out the important consideration that MS and ELISPOT-type methods are fundamentally distinct experimental approaches, reporting different outputs, with different sources of error. Ideally, the approaches should be considered complementary and not opposing. We also note that ARTEMIS analyses of HPV16 E7 with the A2 SCD only identified the 12-mer peptide YMLDLQPETTDL, not a nested 9-mer (YMLDLQPET) that had been identified in a previous MS analysis (56).

The most interesting ARTEMIS result was the identification of the A*11:01-restricted FTSTPPKPK 9-mer which overlaps with a T cell assay identified 10-mer, SASFTSTPPK. However, the ARTEMIS peptide identified by MS was very likely phosphorylated, with over a 90% probability of phosphorylation of the P4 threonine and less than 10% probability on the P3 serine. A recent MS analysis of threonine phosphorylation in LT reported that the threonine residues at the P2 and P4 positions in this peptide can be phosphorylated on native LT (57). However, while phosphorylation on P4 is fully compatible with A*11:01 binding, and phosphorylation on P3 can reasonably be accommodated (e.g., phenylalanine was readily accommodated at P3 (**Figure 7**), phosphorylation on a P2 threonine would be incompatible with A*11:01 binding. Structures of the A11 binding groove (**Figures 10B, C**) show that a phosphothreonine residue is readily accommodated at P4, presented for read-out by a cognate TCR. The apparent steric clash of the phosphoserine at P3 with the A11 groove is “soft” in that the peptide backbone could readily relax to accommodate this substitution; for instance, larger phenylalanine and tyrosine side-chains are readily accommodated at P3 in A11 ARTEMIS results (**Figure 7**). However, the steric clash of the phosphothreonine at P2 is “hard” in that this substitution is unresolvably incompatible with the P2 pocket in the A11 groove. Other nested, A*11:01-restricted peptides were identified by ARTEMIS that were not phosphorylated, indicating that phosphorylation on these sites was not complete. Therefore, ARTEMIS reported presentation of the subset of modified LT peptides derived from this sequence locus consistent with native LT phosphorylation and the known A*11:01 motif, an unexpected outcome that serendipitously raises confidence in ARTEMIS identifications.

While our current MS protocols do not identify peptides with fully elaborated N-glycans, the MSLN proprotein has four potential N-glycan sites, one in the MPF moiety and three in the MSLN moiety. While none of the MSLN sites ended up in an ARTEMIS-identified peptide, one MPF site did: the second asparagine in the HLA-A*02:01-restricted GVLANPPNI 9-mer is in an NIS potential N-glycan site (**Table 2**, **Figure 11**). Since the unglycosylated peptide was observed, the cleavage event/s generating this peptide likely occurred prior to initial co-translational glycosylation in the lumen of the ER, acknowledging that a peptide observed in a pHLA with an *intact* NX^S^/T N-glycan site could be glycosylated on the source protein sequence prior to cleavage or glycosylated in the pHLA complex after loading.

Qualitatively, more MSLN peptides were identified per kDa of protein than for other co-transduced target proteins. Multiple factors were likely in play, for instance protein stability, aborted translation rate, and the degree of post-translational modification than might mask or prevent HLA binding, but the high level of MSLN proprotein expression potentially contributed to the effect: likelihood of a protein contributing at least one peptide to the HLA ligandome has previously been correlated with mRNA expression level (58). In this context, we note that optimized transduction of SCD and target protein potentially leads to overexpression of both species. On the plus side, this increases detection of poorly presented peptides; on the negative side, this may lead to identifying peptides that are not presented physiologically. However, ARTEMIS has been optimized specifically to capture presentomes as fully as possible, in part to enable mechanistic studies of processing and presentation in an experimental context. Identified peptides were also more evenly distributed over the source protein sequence (including the leader peptide) than for LT and achieved coverage of over half the proprotein sequence with only three HLA-A alleles. ARTEMIS-identified MSLN peptides skewed longer than average for the tested alleles, but these analyses also returned thousands of background peptides where the overall length distribution matches reference datasets.

ARTEMIS has been developed into a powerful complementary MS technique for studying multiple aspects of HLA-I antigen processing and presentation and for identifying potentially clinically useful pHLA targets–and can be modularly expanded to additional alleles and target proteins-of-interest. Next-step validation of ARTEMIS involves biochemical and structural corroboration of peptide/HLA binding and confirmation of endogenous T cell responses. Future applications of ARTEMIS include studying ligandome dynamics and responses to intracellular changes and extracellular signals, further definition of allele-specific limiting presentomes, and deeper analyses of the presentation of modified peptides. Since SCDs were successfully expressed for additional alleles, including non-classical HLA molecules, ARTEMIS can likely be widely applied across MHC class I molecules.

## Data Availability Statement

The authors declare that all data supporting the findings of this study are available within the paper. The original mass spectrometry data may be downloaded from MassIVE (http://massive.ucsd.edu) using the identifier MSV000087172.

## Author Contributions

KF and RS designed the study, analyzed results, and wrote the manuscript. LJ performed MS experiments and KF, LJ, CL, M-YB and PG analyzed MS data. AF-G performed statistical analyses. All authors contributed to the article and approved the submitted version.

## Funding

Research reported in this publication was supported by the National Institute of Allergy and Infectious Diseases of the National Institutes of Health under award numbers R01AI121242 and R21AI154874 (RS), a Fred Hutch Joint IRC Pilot Award for New Technology & Data Analysis (AF-G and RS), the National Institutes of Health under award number P30CA015704 (Proteomics Shared Resource of the Fred Hutch/University of Washington Cancer Consortium), and Project Violet (www.projectviolet.org). The M.J. Murdock Charitable Trust funded the acquisition of the Orbitrap Fusion mass spectrometer. The content is solely the responsibility of the authors and does not necessarily represent the official views of the National Institutes of Health.

## Conflict of Interest

The authors declare that the research was conducted in the absence of any commercial or financial relationships that could be construed as a potential conflict of interest.
